# Drug Therapy in Intestinal Flatulence

**Published:** 1911-03

**Authors:** 


### ABSTRACTS

### Drug Therapy in Intestinal Flatulence

Professor Boas (Berlin, Germany), in discussing the various forms of flatulence, their etiology and treatment (*Berliner klinische Wochenschrift,* 1910, No. 3, page 89), states that the leading factor in the treatment of the most common form of flatulence, that of alimentary origin, is, of course, the exclusion of fermentable foods from the diet. Since individuals differ markedly with respect to their digestion of different foods, their tolerance for the various foodstuffs must first be determined. Milk, buttermilk, eggs and rare meats are mentioned among the foods most apt to result in fermentation. Potatoes, too, are frequently responsible for flatulence in individual cases.

Boas has found magnesium salicylate the best antifermentative drug with the particular advantage of not causing constipation. He uses it in doses of 15 to 30 grains three times per day. Where constipation and catarrhal inflammation are associated with the flatulence, small repeated doses of castor oil are recommended.

Magnesium salicylate tablets (Schering) contain 5 grains of pure magnesium salicylate with an addition of a small quantity of cocoa, the latter improving the taste and likewise facilitating ready disintegration.

